# The fibroid phenotype of biological naïve patients with rheumatoid arthritis are less likely to respond to anti-IL-6R treatment

**DOI:** 10.1038/s41598-024-61435-2

**Published:** 2024-05-10

**Authors:** Sofie Falkenløve Madsen, Dovile Sinkeviciute, Christian S. Thudium, Morten A. Karsdal, Anne-Christine Bay-Jensen

**Affiliations:** 1 Department of Biomedical Sciences, University of Copenhagen, Copenhagen, Denmark; 2 ImmunoScience, Nordic Bioscience, Herlev Hovedgade 205, 2730 Herlev, Denmark

**Keywords:** Biomarkers, Rheumatology

## Abstract

Type III collagen gene expression is upregulated in the synovium of patients with rheumatoid arthritis (RA) presenting the fibroid phenotype. The soluble type III collagen formation biomarker, PRO-C3, is known to measure fibrogenesis in fibrotic diseases. In this exploratory study, we aimed to investigate the association between fibrogenesis (PRO-C3) and the disease- and treatment response in patients with RA. We measured PRO-C3 in subsets of two clinical trials assessing the effect of the anti-interleukin-6 (IL-6) receptor treatment tocilizumab (TCZ) as monotherapy or polytherapy with methotrexate. PRO-C3 levels had weak or very weak correlations with the clinical parameters (Spearman’s). However, when the patients were divided into Disease Activity Score-28 groups characterized by the erythrocyte sedimentation rate (DAS28-ESR), there was a statistical difference between the PRO-C3 levels of the different groups (p < 0.05). To determine the response in relation to PRO-C3, a cut-off based on PRO-C3 levels and patients in remission (DAS28-ESR ≤ 2.6) was identified. This showed that a reduction in PRO-C3 after treatment initiation was associated with decreased DAS28-ESR and a higher response rate in patients with low PRO-C3 levels than in those with high PRO-C3 levels. This indicates that a fibrotic component affects the responsiveness of patients.

## Introduction

Rheumatoid arthritis (RA) is a chronic autoimmune disease with a prevalence of 0.5% to 1% worldwide^1,2^. The disease is characterized by synovial membrane inflammation and subsequent joint damage, primarily affecting the small joints of the hands and feet^2,3^. The resulting inflammation and damage manifest as joint pain, swelling, and tenderness^2^. Early diagnosis and prompt treatment are crucial, as it can effectively slow joint damage or destruction and prevent irreversible disabilities^2^. A predictor of joint damage and physical disability in patients with RA is the disease activity score based on 28 joints (DAS28). DAS28 encompasses factors such as the number of swollen and tender joints, levels of acute phase reactants in the blood, and the patient global health assessment. The primary treatment objective in RA is to achieve remission, as indicated by DAS28^4^. Methotrexate (MTX) is typically the first-line treatment^5^. Although MTX is effective for many patients, approximately 43–50% of individuals exhibit an inadequate response^5,6^. Following an inadequate response to MTX, the treatment approach for RA involves augmenting therapy with additional anti-inflammatory drugs or biologics to facilitate remission^4^.

The challenges encountered in determining appropriate treatments can be attributed to the heterogeneous nature of the disease. This heterogeneity has led to the identification of various phenotypes, including the lymphoid, myeloid, and fibroid phenotypes^7–9^. These phenotypes are based on RNA sequencing analyses of biopsies obtained from affected joints^7–10^. As RA is characterized by joint inflammation, the phenotypes assessment also focuses on evaluating inflammatory aspects. Both lymphoid and myeloid phenotypes exhibit upregulated immune responses and proinflammatory genes^8^. By contrast, the fibroid phenotype lacks the proinflammatory component but demonstrates increased expression of genes associated with transforming growth factor-beta (TGF-β) signaling and multiple collagens^8,10^. Dennis et al*.* have demonstrated that specific serum-based biomarkers can assess the treatment response of the lymphoid and myeloid phenotypes. They also found that the lymphoid phenotype has the best response to an anti-interleukin-6 receptor (IL-6R) drug, and the myeloid phenotype has the best response to an anti-tumor necrosis factor alpha (TNF-α) drug^8^. Despite the fibroid phenotype being present in 16–27% of patients, a biomarker capable of predicting treatment response for this phenotype has not yet been identified^7–9^. However, the previous assessments of predicting treatment response have focused on inflammation and not on the major driver of the fibroid phenotype, TGF-β, and upregulation of collagens^8,10^.

The type III collagen (COL3A1) gene expression is a highly upregulated gene in the fibroid phenotype^8,10^. The biomarker PRO-C3 assesses the pro-peptide of the α1(III)-chain, which is released during type III collagen formation and has been established as a reliable predictor of fibrogenesis^11–15^. Type III collagen, hence PRO-C3, are released by fibroblasts, and are not specific to one organ. PRO-C3 levels can predict clinical outcomes and distinguish between stable and diseased patients in various fibrotic conditions, including skin, lung, and liver fibrosis^12–15^. However, its role in RA has not been investigated. Notably, patients with RA in the LITHE study exhibit elevated tissue degradation, evidenced by high levels of matrix metalloproteinase (MMP)-degraded type I, II, III, and IV collagen biomarkers (C1M, C2M, C3M, and C4M, respectively)^16–18^. The tissue degradation is correlated with disease activity and can be suppressed by a combination treatment of tocilizumab (TCZ) and MTX^16–18^. In the AMBITION study, which assessed the effect of TCZ and MTX as monotherapy, the same degradation biomarkers have been investigated. TCZ decreased the C1M, C3M, and C4M levels compared to MTX and placebo after 8 weeks of treatment. MTX decreased C3M and C4M compared to placebo after 8 weeks. The change in C3M and C4M also correlated with DAS28 at all follow-up time points (weeks 8, 16, and 24)^19^. In the RADIATE study, which assessed the effect of TCZ as a polytherapy with MTX, both C3M, and C4M were decreased by TCZ [8 mg/kg] + MTX compared to TCZ [4 mg/kg] + MTX and MTX alone^20,21^.

As PRO-C3 is a predictor of fibrogenesis in other fibrotic diseases, we would like to build the hypothesis that the fibroid phenotype in RA can also be characterized by increased fibrogenesis^11–15^. There is often an imbalance between the formation and degradation of extracellular matrix proteins, such as collagens, when the tissue is fibrotic. As it has previously been shown that TCZ can inhibit the degradation of type III collagen (C3M) in both the AMBITION and RADIATE studies, we hypothesize that TCZ will also affect the formation of type III collagen (PRO-C3)^19–21^. To investigate this hypothesis, we conducted this explorative biomarker sub-study examining the level of fibrogenesis, measured by PRO-C3, in the AMBITION and RADIATE studies. We aimed to explore the association between PRO-C3 levels and DAS28 in relation to both treatment and response.

## Results

### Study demographics

Patients were randomly divided into three treatment groups in the original clinical trials, all with well-balanced demographics and baseline characteristics^22,23^. In this exploratory biomarker sub-study, 342 patients from the AMBITION study and 178 patients from the RADIATE study were included. In the AMBITION study, 306 patients were excluded based on a missing serum sample, and 25 patients were excluded because of missing DAS28 information. In RADIATE, 298 patients were excluded because of a missing serum sample, and 23 patients were based on missing DAS28 information. No statistical differences were observed in the baseline demographics of the three treatment groups within either the AMBITION (Supplementary Table S1) or RADIATE (Supplementary Table S2) studies.

### Study comparison

Differences between the two biomarker sub-studies were compared. Patients in the RADIATE study had a longer median disease duration than those in the AMBITION study (9.5 years vs. 3.2 years, p < 0.001; Table 1). Patients in the RADIATE study exhibited higher levels of health assessment questionnaire-disease index (HAQ-DI), both clinically (ΔHAQ-DI > 0.22) and statistically (p < 0.001; Table 1)^24^. The difference in the HAQ-DI between the two studies was higher in this sub-study than in the original studies, where the difference was 0.20^22,23^. Further, significant differences were observed in pain and patient visual analogue scale (VAS), with higher values in the RADIATE study compared to the AMBITION study. However, these differences did not reach clinical significance (ΔVAS > 9 mm)^25,26^. The baseline characteristics and PRO-C3 levels were similar between the two studies (Table 1).

**Table 1:** Comparisons of baseline demographics of the AMBITION and RADIATE studies.

	AMBITION (n = 342)	RADIATE (n = 178)	Difference	p-value
Age, years	52.0 (16.0)	54.5 (16.5)	–	0.09
Women, n (%)	269 (78.7)	143 (80.3)	–	0.78
BMI	26.6^#^ (6.9)	26.3 (8.1)	–	0.78
**Duration of disease, years**	**3.2 (10.1)**	**9.5 (11.6)**	–	** < 0.001**
DAS28-ESR	6.8 (1.2)	6.9 (1.4)	–	0.39
CRP (mg/dL)	1.7 (3.2)	2.2 (3.5)	–	0.54
ESR (mm/h)	42.0 (29.0)	42.5 (41.5)	–	0.67
**HAQ-DI**	**1.5 (0.8)**	**1.8 (0.8)**	**0.23**^¤^	** < 0.001**
SJC	17.0 (12.0)	18.0 (15.0)	–	0.93
TJC	31.0 (22.0)	31.5 (24.0)	–	0.78
Pain VAS 100 mm	61.0 (30.0)	69.0 (26.8)	8.0^¤¤^	0.01
Physician VAS 100 mm	66.0^##^ (22.0)	70.5 (26.8)	4.5^¤¤^	0.35
Patient VAS 100 mm	67.0 (30.0)	74.0 (27.0)	4.0^¤¤^	< 0.001
PRO-C3 baseline (ng/mL)	12.2^§^ (5.3)	11.8^§^ (5.6)	–	0.52
PRO-C3 follow-up (ng/mL)	11.6^§^ (5.8)	11.1^§^ (6.7)	–	0.82

Values expressed as median (IQR), except when indicated otherwise.

*BMI* body mass index, *DAS28-ESR* disease activity score-28 based on erythrocyte sedimentation rate, *CRP* C-reactive protein, *ESR* erythrocyte sedimentation rate, *HAQ-DI* health assessment questionnaire disease index, *SJC* swollen joint count, *TJC* tender joint count, *VAS* visual analogue scale, *PRO-C3* the pro-peptide of type III collagen.

^#^4 missing BMI values.

^##^1 missing VAS value.

^¤^Clinically relevant HAQ-DI difference: > 0.22^24^.

^¤¤^Clinically relevant VAS score difference: > 9 mm^25,26^.

^§^Missing PRO-C3 values: 7 missing at AMBITION baseline, 36 missing at AMBITION follow-up, 37 missing in RADIATE at baseline and follow-up. The patient demographics were compared with the Mann–Whitney U test with false discovery rate correction.

Significant values are in bold.

### PRO-C3 levels

Serum levels of PRO-C3 were measured at baseline and follow-up (AMBITION, week 8; RADIATE, week 16). No change was observed from baseline to follow-up in PRO-C3 levels within any treatment arms (Supplementary Fig. S1). At baseline, in AMBITION, there was a very weak correlation between PRO-C3 and DAS28-ESR and HAQ-DI, and a weak correlation to C-reactive protein (CRP), erythrocyte sedimentation rate (ESR) and age (p < 0.01; Supplementary Table S3). At baseline in RADIATE, there was only a very weak correlation between PRO-C3 and DAS28-ESR (p < 0.05; Supplementary Table S3). As this study was explorative hypothesis-generating analysis, the data was not adjusted to the identified correlations.

The patients included in the studies had active RA at baseline and exhibited moderate to high DAS28-ESR (DAS28 > 3.2). At follow-up (AMBITION, week 8; RADIATE, week 16), the DAS28-ESR information was available for 306 patients in AMBITION and 141 patients in RADIATE. Thus, the DAS28-ESR information for 36 and 37 patients, respectively, was unavailable, and these patients were not included in the follow-up assessments.

At follow-up, the DAS28-ESR of the patients had improved, and there were now patients in all four DAS28-ESR groups. To explicate this outcome further, we subdivided patients into the four DAS28-ESR groups: remission (DAS28 ≤ 2.6), low DAS (2.6 < DAS28 ≤ 3.2), moderate DAS (3.2 < DAS28 ≤ 5.1) and high DAS (DAS28 > 5.1). The PRO-C3 levels differed between the four DAS28 groups at follow-up, with patients in remission showcasing the lowest levels of PRO-C3 (AMBITION: p = 0.002; RADIATE: p = 0.045, Table 2). Similar differences were observed when the patients were only subdivided into remission (DAS28 ≤ 2.6) and disease activity (DAS28 > 2.6) (AMBITION: p = 0.017; RADIATE: p = 0.023, Table 2).

**Table 2:** Comparison of PRO-C3 levels at follow-up with DAS28-ESR response groups.

	AMBITION (week 8) (n = 306)		RADIATE (week 16) (n = 141)	
	Patients, n (%)	PRO-C3 ng/mL, mean [CI]	Patients, n (%)	PRO-C3 ng/mL, mean [CI]
DAS28-ESR groups				
Remission (DAS28 ≤ 2.6)	26 (8.5)	10.6 [8.8;12.3]	12 (8.5)	9.5 [7.0;12.1]
Low DAS (2.6 < DAS28 ≤ 3.2)	12 (3.9)	10.8 [8.1;13.4]	8 (5.7)	10.7 [8.1;13.2]
Moderate DAS (3.2 < DAS28 ≤ 5.1)	100 (32.7)	11.8 [10.9;12.6]	63 (44.7)	12.4 [11.0;13.8]
High DAS (DAS28 > 5.1)	168 (54.9)	13.7 [12.7;14.7]	58 (41.1)	14.7 [12.6;16.9]
p-value		**0.002**		**0.045**
Remission vs. Disease activity				
Remission (DAS28 ≤ 2.6)	26 (8.5)	10.6 [8.8;12.3]	12 (8.5)	9.5 [7.0;12.1]
Disease activity (DAS28 > 2.6)	280 (91.5)	12.9 [12.2;13.6]	129 (91.5)	13.3 [12.2;14.5]
p-value		**0.017**		**0.023**

The PRO-C3 levels of the DAS groups were compared with the Kruskal–Wallis test and corrected for multiple comparisons when appropriate. Only patients with PRO-C3 values at follow-up were used; thus, the 36 patients missing in AMBITION and the 37 patients missing in RADIATE at follow-up were not included.

*PRO-C3* type III collagen formation biomarker, *DAS28* disease activity score 28 with erythrocyte sedimentation rate, *CI* confidence interval. Significant values are in bold.

### Association and prediction of treatment response

The analysis of PRO-C3’s association with and prediction of treatment response included only patients receiving active treatment at follow-up; 262 patients from AMBITION and 141 from RADIATE were included. Cut-off values were determined using receiver operating characteristic (ROC) curve analysis, with the treatment response defined as disease remission (DAS28 ≤ 2.6) and treatment non-response as active disease (DAS28 > 2.6) at follow-up and PRO-C3 levels at follow-up as the predictor. The change in DAS28-ESR at follow-up was independent of DAS28-ESR at baseline, where all patients had either moderate or high DAS28-ESR (DAS28 > 3.2).

**Figure 1:**
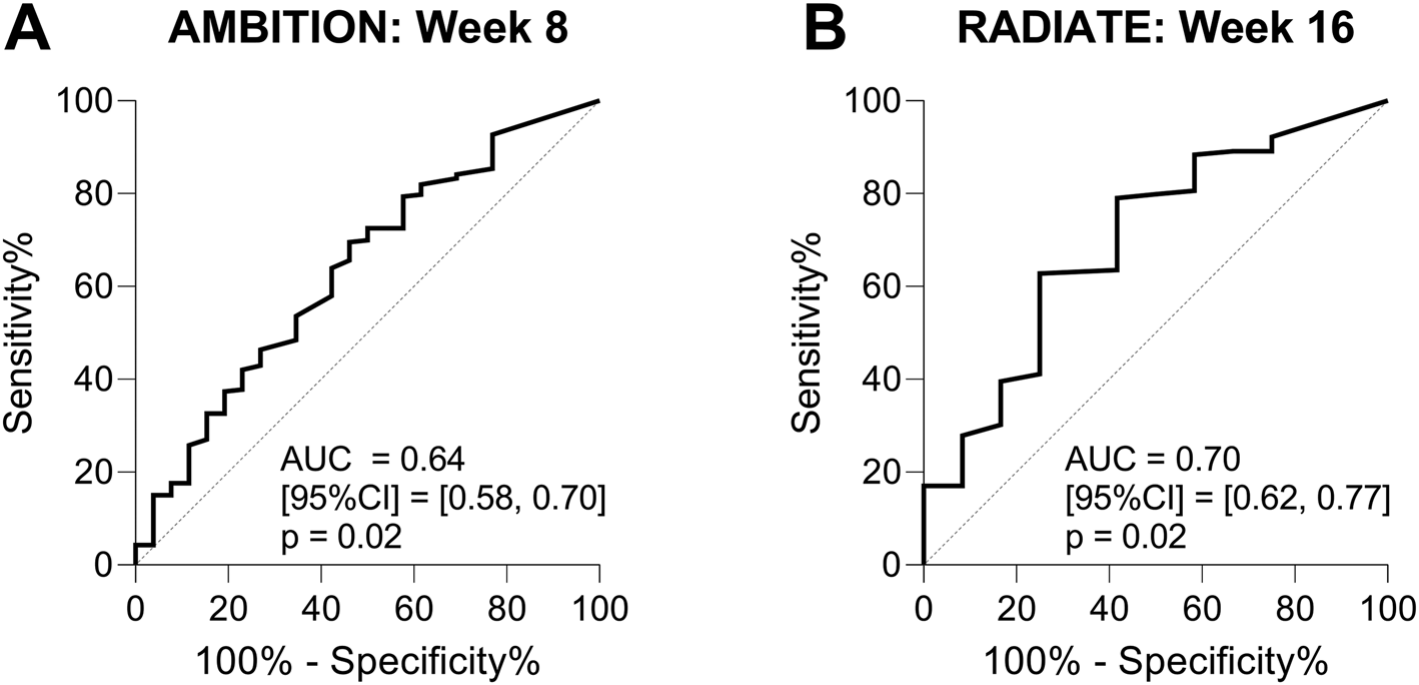
Performance of PRO-C3 levels as a predictor of DAS28-ESR outcome. Results are shown as an area under the receiver operating characteristic curve (AUC), labeled with 95% confidence intervals (95% CI). (**A**) ROC curve of AMBITION where 26 patients achieved remission after 8 weeks of treatment: AUC = 0.64, p = 0.02. Sensitivity of 69.5% and specificity of 53.8%, leading to a cut-off > 9.5 ng/mL PRO-C3. The analysis only included patients on active treatment. (**B**) ROC curve of RADIATE, where 12 patients achieved remission after 16 weeks of treatment: AUC = 0.70, p = 0.02. Sensitivity of 62.8%, and specificity of 75.0%, leading to a cut-off > 9.8 ng/mL PRO-C3.

In the AMBITION study, 26 (9.9%) out of 262 patients achieved disease remission after 8 weeks of treatment. This resulted in a 9.5 ng/mL cut-off value for PRO-C3 (AUC = 0.64, p = 0.02, Fig. 1A). In the RADIATE study, 12 out of 141 patients (8.5%) achieved disease remission after 16 weeks of treatment. This led to a 9.8 ng/mL cut-off value for PRO-C3 (AUC = 0.70, p = 0.02, Fig. 1B). Of the 26 responders in AMBITION at week 8, 25 patients were treated with TCZ and one with MTX (Supplementary Table S4). The 12 responders at week 16 in RADIATE were all in TCZ [8 mg/kg] + MTX treatment (Supplementary Table S5).

Treatment response rates at the first follow-up time point were evaluated (AMBITION, week 8, and RADIATE, week 16) and revealed that patients with low PRO-C3 levels exhibited a higher response rate than those with high PRO-C3 levels (AMBITION 15.5% vs. 7.3%; RADIATE 13.0% vs. 5.7%; Table 3). The response rates remain higher in the low PRO-C3 group compared to the high PRO-C3 group throughout all follow-up time points in both AMBITION (Week 16: 21.3% vs. 11.0%. Week 24: 31.2% vs. 21.7%) and RADIATE (Week 24: 25.0% vs. 12.9%) (Table 3). The distribution between treatments within the low/high PRO-C3 groups can be found in Supplementary Tables S6 and S7.

**Table 3:** Response rates of low/high PRO-C3 patients.

	PRO-C3 values (ng/mL)	Responders, n DAS28 ≤ 2.6	Non-responders, n DAS28 > 2.6	Response rate, %	Odds ratio, OR [95% CI]	p-value
AMBITION						
Week 8 (n = 262)	Low (n = 84)	13	71	15.5	2.3 [1.0, 5.3]	**0.05**
	High (n = 178)	13	165	7.3		
Week 16 (n = 252)	Low (n = 80)	17	63	21.3	2.2 [1.1; 4.3]	**0.04**
	High (n = 172)	19	153	11.0		
Week 24 (n = 243)	Low (n = 77)	24	53	31.2	1.6 [0.9; 3.0]	0.11
	High (n = 166)	36	130	21.7		
RADIATE						
Week 16 (n = 141)	Low (n = 54)	7	47	13.0	2.4 [0.7; 8.1]	0.21
	High (n = 87)	5	82	5.7		
Week 24 (n = 102)	Low (n = 40)	10	30	25.0	2.3 [0.9; 6.6]	0.18
	High (n = 62)	8	54	12.9		

The patients were divided into four groups based on whether they had low or high PRO-C3 at the first follow-up (AMBITION, week 8, cut-off 9.5 ng/mL; RADIATE, week 16, cut-off 9.8 ng/mL) and whether they were a responder (DAS28 ≤ 2.6) or a non-responder (DAS28 > 2.6) at the different follow-up times. Only patients on active treatment and with DAS28 information available were used for this calculation. In AMBITION, 262 patients were included at week 8, 10 patients were lost to follow-up in week 16, and 9 more were lost in week 24. In RADIATE, 141 patients were included at week 16, and 39 patients were lost to follow-up at week 24. The response rate and odds ratio were calculated using 2 × 2 contingency tables with Fisher’s exact test.

Significant values are in bold.

In the AMBITION study, patients with high levels of PRO-C3 at week 8 were more likely to be non-responders at weeks 8 and 16, compared to patients with low PRO-C3 levels (odds ratios (ORs) of 2.3 and 2.2, respectively; Table 3). The trend remained at week 24 but was not significant (OR 1.6; Table 3). In the RADIATE study, patients with high levels of PRO-C3 at week 16 were more likely to be non-responders at weeks 16 and 24, compared to patients with high levels (ORs of 2.4 and 2.3, respectively); however, this was not statistically significant due to the low number of responders (Table 3).

## Discussion

In this explorative and hypothesis-generating biomarker sub-study of the AMBITION and RADIATE studies, we investigated the association between fibrogenesis and disease activity in relation to treatment and response in patients with RA. Our results show that high levels of PRO-C3 are associated with high disease activity after treatment initiation and a lower likelihood of treatment response. Historically, inflammation has been the primary focus in RA, although previous studies support the existence of fibroid phenotype and fibrolysis in patients with RA^7,8,19–21,27,28^. Thus, the presence and formation of a fibrotic component have not been investigated in detail. Biopsies from affected joints in patients with RA have revealed different phenotypes, such as the lymphoid, myeloid, and fibroid phenotypes^7–10^. Although biopsies are interesting in research, they are not feasible for use in the clinical assessment of RA, as non-invasive methods, such as clinical characteristics and radiological or serological manifestations, are available^29^. Blood-based biomarkers are also non-invasive, which might add relevant information about the phenotypes, as indicated in this study.

The biomarker PRO-C3 is a well-known biomarker of fibrogenesis^12,30^. In liver fibrosis, PRO-C3 levels increase with the progression of fibrosis, and it has been successfully used as a non-invasive biomarker to assess the liver fibrosis stage, which is usually assessed with a biopsy^12,30^. PRO-C3 has also shown associations with the non-invasive modified Rodnan skin score in systemic sclerosis (SSc), another fibro-inflammatory disease^31^. The COL3A1 gene, which encodes type III collagen, has been shown to be upregulated in the fibroid phenotype in RA^8,10^. As PRO-C3 measures the α1(III)-chain, increased PRO-C3 could indicate the presence of the fibroid phenotype^12^. PRO-C3 has not previously been investigated in RA, although patients with RA have upregulated tissue degradation^16–18^. In AMBITION, TCZ has been shown to decrease C1M, C3M, and C4M levels compared to MTX and placebo, while MTX decreased C3M and C4M compared to placebo. The decrease of C3M and C4M by MTX also correlated with DAS28^19^. In RADIATE, TCZ [8 mg/kg] + MTX also decreased C3M and C4M compared to TCZ [4 mg/kg] + MTX and MTX alone^20,21^.

The healthy reference level of PRO-C3 has been established in several studies, with a reported median concentration of 8.9 ng/mL^30^ and mean concentrations that range from 10 to 14.2 ng/mL^14,15,32^. The same studies assessed diseased patients with average PRO-C3 levels between 9.5 and 20.8 ng/mL^14,15,30,32^. In this study, the distribution of PRO-C3 levels in patients with RA is in the range of 6.1–80.2 ng/mL, with a median between 11.1 and 12.2 ng/mL, depending on the time point and study. Thus, the median PRO-C3 concentration of patients with RA is higher than that of healthy subjects^30^. In both SSc and idiopathic pulmonary fibrosis (IPF), PRO-C3 levels have been demonstrated to be predictive, as the levels can differentiate between stable and progressive disease stages^14,15^. Patients with progressive SSc and IPF have high PRO-C3 levels (14–17 ng/mL), while stable patients have low levels (11–15 ng/mL) that are close to those in the healthy controls^14,15^. Similar assessments can be made in patients with RA, between patients in remission versus active disease. In this study, patients in remission had average PRO-C3 levels of 9.5–10.6 ng/mL, close to the levels found in healthy controls and in patients with stable SSc and IPF^14,15,30,32^. Meanwhile, patients with active disease (DAS28 > 2.6) had average PRO-C3 levels closer to the levels observed in progressive patients (12.9–13.3 ng/mL)^14,15^. The high levels of PRO-C3, thus fibrogenesis, indicate the presence of the fibroid phenotype, similar to increased fibrogenesis in liver fibrosis and SSc^12,15,30^. In both AMBITION and RADIATE, patients with RA in remission had significantly lower PRO-C3 levels than patients with active disease (Table 2). Similar results were obtained when the patients were assessed in the four DAS28-ESR groups (Table 2).

In the present study, the biomarker levels of PRO-C3 were not affected by the individual treatment arms. Instead, the PRO-C3 levels followed the disease activity, as decreasing fibrogenesis (PRO-C3) followed decreasing DAS28-ESR (Supplementary Fig. S1, Table 2). This means that a specific treatment does not modulate PRO-C3 but instead follows the improvement of disease activity, thus the patient’s response to the treatment they receive. In the assessment of the association and predictive capacity of PRO-C3 in relation to disease remission (DAS28-ESR ≤ 2.6), it was found that patients with low levels of PRO-C3 had a better response rate to treatment than patients with high levels of PRO-C3 (Table 3).

Typically, the assessment of fibrogenesis in RA has primarily focused on the development of interstitial lung disease (RA-ILD). Several biomarkers have been proposed to predict which patients will develop RA-ILD, while none specifically focus on fibrosis or fibrogenesis^33–35^. PRO-C3 is a predictor of progression in other fibrotic diseases; thus, it may have the potential to predict progression in RA and RA-ILD^14^. PRO-C3 levels can indicate systemic levels of fibrogenesis, which can originate from various tissues, including the lungs, liver, and joints. Although PRO-C3 levels in patients with RA have a wide span, fibrogenesis appears to be more prevalent in patients with high disease activity (Table 2). As PRO-C3 levels can predict disease progression in both SSc and IPF, this might also be true for patients with RA with high PRO-C3, thus high disease activity. We speculate that high PRO-C3 levels in patients with RA might be a factor in developing or detecting ILD in patients with RA. However, there is no available information about the progression of ILD in the patients used in this study; thus, further investigations are needed to explore the association between PRO-C3 and RA-ILD development.

The potential of PRO-C3 levels to distinguish between remission and disease activity in patients with RA showed an area under the receiver operating characteristic (AUROC) of 0.64–0.70. The AUROC values indicate moderate discriminatory ability; further improvement could be achieved by combining PRO-C3 with other known biomarkers or clinical factors. The combination could be with biomarkers already used in the clinic, such as rheumatoid factor and anti-citrullinated protein antibody.

The limitations of this study include that the sample size in this exploratory sub-study was smaller than intended in the original trials, as only patients with available serum samples and DAS28-ESR information were included. The trials were also independent of each other and focused on different subgroups of patients, which were also different in disease duration and HAQ-DI, thus making them not directly comparable. Moreover, the ideal study design would have included a healthy control group for comparison. A comparison with a biopsy or ultrasound assessments would be optimal to directly compare the fibrogenesis detected in blood and the potential fibrogenesis in the joints of RA. However, synovial biopsies and ultrasound assessments were not available in either of the assessed studies. This study was exploratory and hypothesis-generating, thus hypothesis-testing studies are needed. In this study, we assessed potential confounding factors, but due to the exploratory state of the study, we did not adjust for them. Future hypothesis-testing studies should also assess, and potentially adjust for confounding factors. Future studies should also investigate when PRO-C3 levels start to increase and whether they can serve as predictive biomarkers for the onset of RA. Validation studies are also necessary to determine whether different PRO-C3 cut-offs are needed for early- and late-stage RA or whether a universal cut-off can be applied to all patients with RA.

In conclusion, patients with RA exhibiting high levels of measurable fibrogenesis, as measured by PRO-C3, are less likely to respond to TCZ compared to patients with low levels. This indicates that a fibrotic component affects the responsiveness to anti-inflammatory treatment in patients with RA. High fibrogenesis also indicates the presence of the fibroid phenotype; thus, we speculate that these patients would potentially benefit from treatments that target fibrogenesis instead or together with inflammation.

## Methods

### Patients

The AMBITION (NCT00109408) and RADIATE (NCT00106522) studies enrolled adult patients aged 18 years or older with moderate to severe active RA. Active RA was defined by a swollen joint count (SJC) of 6 or more, tender joint count (TJC) of 8 or more, and elevated levels of C-reactive protein (CRP) (> 1 mg/dL) or erythrocyte sedimentation rate (ESR) (> 28 mm/h)^22,23^.

In the AMBITION study, patients had to have active RA for at least 3 months to be included. They were allowed to receive a stable dose of oral glucocorticoids and nonsteroidal anti-inflammatory drugs (NSAIDs) if the dose had been stable for at least 6 weeks before the start of the study. Patients were excluded if they had uncontrolled medical conditions, previous unsuccessful treatment with anti-TNF-α or MTX, or had received MTX within the 6 months preceding randomization. Patients who temporarily discontinued MTX or anti-TNF-α treatment for reasons other than efficacy could still be included in the study^22^. Consequently, the patients were categorized as biological-naïve patients.

In the RADIATE study, patients had to have active RA for at least 6 months to be included. The patients had either failed to respond to one or more anti-TNF-α treatments or had experienced intolerance to such treatments within the past year. They had to be treated with MTX for a minimum of 12 weeks before baseline, with a stable dose for the last 8 weeks. Patients in the RADIATE study were also permitted to use oral glucocorticoids and NSAIDs during the study. Before receiving the study medication, they had to discontinue active treatments and all disease-modifying antirheumatic drugs (DMARDs) other than MTX. Patients were excluded if they had received cell-depleting agents or had uncontrolled medical conditions^23^.

### Study protocols

**Figure 2:**
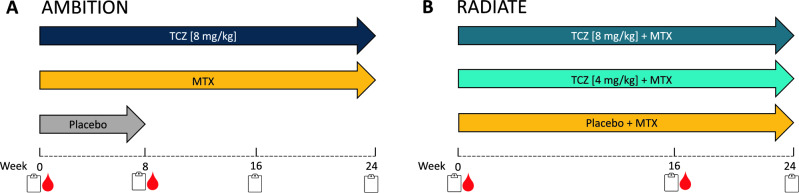
Study design of the AMBITION and RADIATE studies. The arrows indicate the three treatment arms of (**A**) AMBITION and (**B**) RADIATE. The red blood drop represents the visits/week where serum was collected, and the notepad represents when clinical information was collected. *MTX *methotrexate, *TCZ *tocilizumab.

The AMBITION study is a phase III clinical trial conducted over 24 weeks. The study involved 673 patients who were randomized into three treatment groups. The first group received intravenous (IV) TCZ monotherapy infusions of an 8 mg/kg dose every 4 weeks. The second group received MTX monotherapy, administered orally weekly, together with folate. The third group received a placebo for the first 8 weeks, followed by TCZ at a dose of 8 mg/kg every 4 weeks for the remaining 16 weeks of the study. Patients in the placebo group were able to receive rescue treatment of TCZ 8 mg/kg if they experienced 20% worsening in SJC and TJC. When changing to rescue treatment, no further clinical information was used. The study was conducted as a double-blind, double-dummy, parallel-group design^22^. Clinical information was available from baseline, week 8, week 16, and week 24; serum samples were available from baseline and week 8 (Fig. 2A).

The RADIATE study is a phase III clinical trial conducted over 24 weeks. The study involved 499 patients who were randomized into three treatment groups. The first group received TCZ 8 mg/kg IV every 4 weeks, the second group received TCZ 4 mg/kg IV every 4 weeks, and the third group received placebo IV every 4 weeks. All groups received IV in combination with stable MTX. In all cases of treatment failure (defined as less than 20% improvement in both SJC and TJC at week 16), rescue therapy with TCZ 8 mg/kg IV in combination with MTX was offered. After changing to rescue therapy, clinical information on those patients was no longer used. The study was conducted as a double-blind, placebo-controlled, parallel-group design^23^. Clinical information was available from baseline, week 16, and 24, and serum samples were available from baseline and week 16 (Fig. 2B).

Ethics committees and regulatory authorities approved both study protocols, and written informed consent was obtained from each participant prior to their involvement. The studies were conducted in accordance with the principles outlined in the Declaration of Helsinki^22,23^. This biomarker sub-study was approved by Nordic Bioscience A/S ethics committee.

### Biomarker study subsets

**Figure 3:**
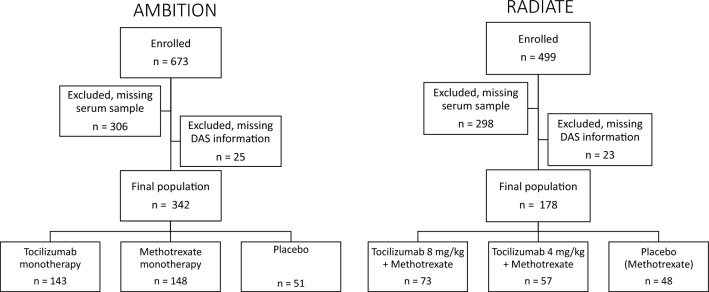
Overview of patient exclusion and inclusion of the AMBITION and RADIATE studies. The patients were excluded based on missing serum samples and/or missing DAS28-ESR information. The final population in this biomarker sub-study is shown, including the separation of the patients in the three treatment arms of both studies.

In this biomarker sub-study, patients were included if there was an available serum sample and if the DAS28-ESR information was available for both baseline and the first follow-up timepoint. In AMBITION, 306 patients were excluded because of missing serum samples at both baseline and follow-up and in RADIATE, 298 patients were excluded. Additionally, 25 patients in AMBITION and 23 patients in RADIATE were excluded based on missing DAS28-ESR information at either baseline or follow-up (Fig. 3). In the AMBITION study, four patients had missing body mass index (BMI), and one patient was missing physician visual analogue scale (VAS) information at baseline. Despite these missing data points, these patients were still included in the biomarker sub-study, as the primary focus was assessing the biomarker outcome. The final population in the sub-study consisted of 342 patients from the AMBITION study and 178 patients from the RADIATE study (Fig. 3).

### Enzyme-linked immunosorbent assay

Type III collagen formation was measured in serum using the validated NordicPRO-C3™ competitive enzyme-linked immunosorbent assay (ELISA), following the manufacturer’s instructions (cat. No. 1700)^12^. The measurements were quality-controlled with appropriate calibrators, and a calibration curve was generated using a four-parameter logistic model. The calibration curve was accepted within the analytical measurement range (MR), with a coefficient of variance (CV) below 10% and a relative error below 15%. Sample measurements were performed in duplicates and were accepted within the MR and with a CV below 15%. Samples below the lower limit of quantitation (LLOQ) were assigned the value of LLOQ: 6.1 ng/mL.

### Statistical analyses

In this explorative and hypothesis-generating post hoc biomarker analysis, 652 patients were excluded, and 540 patients were included.

As only subsets of patients from the original clinical studies were included in this analysis, the treatment groups within each trial were compared to ensure they were still comparable at baseline. The treatment groups within each clinical trial were compared at baseline using the Kruskal–Wallis test and adjusted for multiple comparisons using the false discovery rate (FDR). For categorical variables, the Mann–Whitney U test was used. Additionally, patient demographics between the two clinical trials were compared using the Mann–Whitney U test, with FDR correction applied to identify any differences. The difference between the assessment of the health assessment questionnaire-disability index (HAQ-DI) and VAS should be above the clinical significance thresholds established in previous studies for it to have clinical significance (ΔHAQ-DI > 0.22; ΔVAS > 9 mm)^24–26^.

The correlation between PRO-C3 levels at baseline and clinical parameters was assessed using Spearman’s rank correlation. This analysis aimed to investigate whether the biomarker values potentially needed to be adjusted for any clinical parameters. As this was an exploratory analysis, no adjustments were made, due to the risk of overfitting the model to the specific data set.

A mixed-effects model adjusted for FDR was employed to compare biomarker levels at different time points. The biomarker levels of individual treatment groups were compared between time points and treatment groups at each time point. Statistics and graphical illustrations for this were performed using Graph Pad Prism version 9.5 for Windows (GraphPad Software, San Diego, CA, USA; available from www.graphpad.com).

To assess the association and predictive ability of PRO-C3, cut-offs were identified using the area under the receiver operating characteristic (AUROC) curve analysis. The DAS28-ESR levels were used as the outcome, with DAS28-ESR ≤ 2.6 indicating treatment responders (remission) and DAS28-ESR > 2.6 indicating non-responders (active disease). The Youden index was utilized to determine the optimal cut-off using MedCalc Statistical Software version 19.3 (MedCalc Software 19.3, Ostend, Belgium. Available from: https://www.medcalc.org, 2020). Logistic regressions assessed the independence of DAS28-ESR values and groups to the PRO-C3 cut-off levels. Response rates and odds ratios (OR) were calculated using 2 × 2 contingency tables with Fisher’s exact test.

A significance level of p ≤ 0.05 was employed, and asterisks were used to denote the level of *p < 0.05, **p < 0.01, ***p < 0.001, and ****p < 0.0001. Unless otherwise stated, statistical analysis was conducted in R Studio version 4.2.1 (RStudio Team. RStudio: Integrated Development Environment for R. RStudio, PBC, Boston, MA; available from http://www.rstudio.com).

### Supplementary Information

Supplementary Information.

## Data Availability

The data supporting this study’s findings is available from the corresponding author upon request.
