# Supplementary material for: The fibroid phenotype of biological naïve patients with rheumatoid arthritis are less likely to respond to anti-IL-6R treatment
Source: Sci Rep. 2024 May 10;14:10751. doi: 10.1038/s41598-024-61435-2 (PMC11087519; doi:10.1038/s41598-024-61435-2)
Supplement: Supplementary file 1 — Supplementary Information. [file 41598_2024_61435_MOESM1_ESM.pdf]

Supplementary information

This file contains supplementary material.

Study demographics

Table S1: *AMBITION patient demographics at baseline within the treatment groups*

	**TCZ [8 mg/kg]** (n=143)		**MTX** (n=148)		**Placebo** (n=51)		**p-value**
Age, years	51.0	(16.0)	52.0	(17.0)	52.0	(16.5)	*0.94*
Women, n (%)	117	(81.8)	113	(76.4)	39	(76.5)	*0.78*
BMI	# 26.8	(7.0)	# 26.1	(6.7)	27.3	(7.2)	*0.72*
Duration of disease, years	2.8	(9.1)	3.4	(10.3)	3.1	(12.9)	*0.94*
DAS28	6.74	(1.2)	6.8	(1.2)	7.0	(1.1)	*0.94*
CRP, mg/dL	1.7	(3.4)	2.2	(3.1)	1.6	(1.8)	*0.31*
ESR, mm/h	41.0	(28.5)	43.5	(31.5)	42.0	(27.5)	*0.94*
HAQ-DI	1.5	(0.9)	1.6	(0.9)	1.4	(0.5)	*0.31*
SJC	16.0	(11.0)	17.5	(10.5)	20.0	(17.0)	*0.36*
TJC	30.0	(23.0)	31.0	(21.3)	33.0	(19.5)	*0.78*
Pain VAS 100 mm	61.0	(30.5)	61.5	(28.3)	56.0	(34.5)	*0.78*
Physician VAS 100 mm	63.0	(22.5)	## 66.0	(21.5)	73.0	(22.5)	*0.31*
Patient VAS 100 mm	69.0	(31.0)	65.0	(30.3)	67.0	(28.0)	*0.94*

Values are median (IQR), except when indicated otherwise. # Missing BMI values, TCZ: 3 missing values, MTX: 1 missing value, ## 1 missing Physician VAS value. CRP: C-reactive protein; ESR: erythrocyte sedimentation rate; HAQ-DI: health assessment questionnaire disease index; SJC: swollen joint count; TJC: tender joint count; VAS: visual analog scale. The treatment groups were compared with the Kruskal-Wallis test and corrected for multiple comparisons. Categorical variables were compared with a Mann-Whitney U test. A total of 342 patients were included.

Table S2: *RADIATE patient demographics at baseline within the treatment groups*

	**TCZ [8 mg/kg]** (n=73)		**TCZ [4 mg/kg]** (n=57)		**MTX** (n=48)		**p-value**
Age, years	55.0	(15.0)	53.0	(20.0)	56.5	(16.3)	*0.86*
Women, n (%)	63	(86.3)	44	(77.2)	36	(75.0)	*0.78*
BMI	26.1	(8.3)	26.8	(7.2)	25.7	(9.8)	*0.78*
Duration of disease, years	11.0	(13.6)	9.0	(10.4)	7.3	(15.2)	*0.78*
DAS28	6.9	(1.4)	7.0	(1.2)	6.7	(1.3)	*0.96*
CRP, mg/dL	2.2	(3.4)	2.2	(3.4)	1.9	(3.5)	*0.96*
ESR, mm/h	40.0	(40.0)	42.0	(41.0)	46.0	(42.0)	*0.96*
HAQ-DI	1.9	(0.9)	1.8	(0.8)	1.6	(0.9)	*0.80*
SJC	16.0	(12.0)	19.0	(13.0)	16.0	(17.0)	*0.78*
TJC	31.0	(24.0)	32.0	(24.0)	33.0	(24.0)	*0.96*
Pain VAS 100 mm	69.0	(25.0)	69.0	(26.0)	62.5	(31.3)	*0.88*
Physician VAS 100 mm	70.0	(26.0)	72.0	(23.0)	70.0	(27.0)	*0.96*
Patient VAS 100 mm	74.0	(26.0)	77.0	(32.0)	72.0	(21.3)	*0.88*

Values are median (IQR), except when indicated otherwise. CRP: C-reactive protein; ESR: erythrocyte sedimentation rate; HAQ-DI: health assessment questionnaire disease index; SJC: swollen joint count; TJC: tender joint count; VAS: visual analog scale. The treatment groups were compared with the Kruskal-Wallis test and corrected for multiple comparisons. Categorical variables were compared with a Mann-Whitney U test. A total of 178 patients were included.

Biomarker values

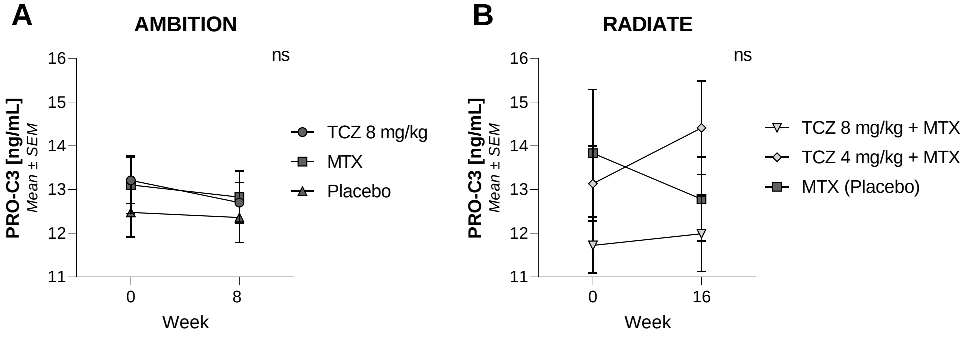

Figure S1: Type III collagen formation (PRO-C3) levels in AMBITION and RADIATE. *The type III collagen formation in ng/mL at baseline and follow-up (A) AMBITION and (B) RADIATE. The individual treatments were compared between the timepoints, and the different treatments was compared at each timepoint with a mixed-effect analysis corrected for multiple comparisons with FDR. Data are presented as mean ± standard error of the mean (SEM).*

Spearman's correlation

To assess any relationship between the biomarker PRO-C3 and the clinical parameters, Spearman's rank correlation was computed for baseline measurements of PRO-C3 and baseline parameters. The correlation or strength of association is divided into groups of very weak, weak, moderate, strong, and very strong (0.0-0.19, 0.2-0.39, 0.40-.059, 0.6-0.79, 0.8-1, respectively).

Table S3: Spearman's correlation of baseline parameters to PRO-C3. *The clinical parameters of AMBITION only have a very weak correlation (0.0-0.19) except age, CRP and ESR with a weak correlation (0.2-0.39) with PRO-C3. The clinical parameters of RADIATE all have a very weak correlation (0.0-0.19) with PRO-C3 at baseline.*

	**AMBITION**		**RADIATE**	
	PRO-C3	p	PRO-C3	p
Age, years	0.25	***	0.08	
BMI	-0.01		0.00	
Duration of disease	-0.10		0.00	
DAS28-ESR	0.17	**	0.19	*
CRP	0.21	***	-0.01	
ESR	0.20	***	0.12	
HAQ-DI	0.18	**	0.02	
SJC	0.05		0.15	
TJC	0.07		0.15	
Pain VAS 100 mm	0.08		0.00	
Physician VAS 100 mm	0.08		0.12	
Patient VAS 100 mm	0.05		0.02	

CRP: C-reactive protein; ESR: erythrocyte sedimentation rate; HAQ-DI: health assessment questionnaire disease index; SJC: swollen joint count; TJC: tender joint count; VAS: visual analog scale.

Treatment response

An overview of the response within the different treatment groups of AMBITION can be found in Table S4 and RADIATE in Table S5. An overview of the response within the low/high PRO-C3 groups and treatment groups can be found in Tables S6 and S7.

Table S4: Treatment response of AMBITION. *The number of patients who were responders (DAS28 ≤2.6) and non-responders (DAS28 >2.6) within the tocilizumab [8 mg/kg] (TCZ) and methotrexate (MTX) treatment groups.*

**AMBITION**		**Responders, n**			**Non-responders, n**		
	**Total**	**Total**	**TCZ**	**MTX**	**Total**	**TCZ**	**MTX**
**Week 8**	262	26	25	1	236	104	132
**Week 16**	252	36	30	6	216	97	119
**Week 24**	243	60	44	16	183	79	104

Table S5: Treatment response of RADIATE. *The number of patients who were responders (DAS28 ≤2.6) and non-responders (DAS28 >2.6) within the three treatment groups: tocilizumab [8 mg/kg] (TCZ), TCZ [4 mg/kg] and methotrexate (MTX).*

**RADIATE**		**Responders, n**				**Non-responders, n**			
	**Total**	**Total**	**TCZ [8 mg/kg] + MTX**	**TCZ [4 mg/kg] + MTX**	**Placebo + MTX**	**Total**	**TCZ [8 mg/kg] + MTX**	**TCZ [4 mg/kg] + MTX**	**Placebo + MTX**
**Week 16**	141	12	12	0	0	129	43	47	39
**Week 24**	102	18	15	3	0	84	34	31	19

Table S6: Treatment response of AMBITION. *The number of patients who were responders (DAS28 ≤2.6) and non-responders (DAS28 >2.6) within the tocilizumab [8 mg/kg] (TCZ) and methotrexate (MTX) treatment groups within the low/high PRO-C3 groups.*

**AMBITION**	**PRO-C3 values (ng/mL)**	**Responders, n**			**Non-responders, n**		
		**Total**	**TCZ**	**MTX**	**Total**	**TCZ**	**MTX**
**Week 8**	**Low (<9.5)**	13	13	0	71	26	45
	**High (>9.5)**	13	12	1	165	78	87
**Week 16**	**Low (<9.5)**	17	14	3	63	24	39
	**High (>9.5)**	19	16	3	153	73	80
**Week 24**	**Low (<9.5)**	24	17	7	53	19	34
	**High (>9.5)**	36	27	9	130	60	70

Table S7: Treatment response of RADIATE. *The number of patients who were responders (DAS28 ≤2.6) and non-responders (DAS28 >2.6) within the three treatment groups: tocilizumab [8 mg/kg] (TCZ), TCZ [4 mg/kg] and methotrexate (MTX) within the low/high PRO-C3 groups.*

RADIATE	**PRO-C3 values (ng/mL)**	**Responders, n**				**Non-responders, n**			
		**Total**	**TCZ [8 mg/kg] + MTX**	**TCZ [4 mg/kg] + MTX**	**Placebo + MTX**	**Total**	**TCZ [8 mg/kg] + MTX**	**TCZ [4 mg/kg] + MTX**	**Placebo + MTX**
Week 16	**Low (<9.8)**	7	7	0	0	47	15	16	16
	**High (>9.8)**	5	5	0	0	82	28	31	23
Week 24	**Low (<9.8)**	10	8	2	0	30	12	11	7
	**High (>9.8)**	8	7	1	0	54	22	20	12
